# Fabrication of Zn-Cu-Ni Ternary Oxides in Nanoarrays for Photo-Enhanced Pseudocapacitive Charge Storage

**DOI:** 10.3390/nano12142457

**Published:** 2022-07-18

**Authors:** Ruitong Xu, Jun Pan, Bo Wu, Yangguang Li, Hong-En Wang, Ting Zhu

**Affiliations:** 1 School of Physics and Electronic Information, Yunnan Normal University, Kunming 650500, China; 2290781440@qq.com (R.X.); 2436391230@qq.com (Y.L.); hongen.wang@outlook.com (H.-E.W.); 2 School of Materials Science & Engineering, Central South University, Changsha 410083, China; 964229306@qq.com (J.P.); 952068584@qq.com (B.W.); 3 Yunnan Key Laboratory of Optoelectronic Information Technology, School of Physics and Electronic Information, Yunnan Normal University, Kunming 650500, China; 4 Key Laboratory of Advanced Technique & Preparation for Renewable Energy Materials, Ministry of Education, Yunnan Normal University, Kunming 650500, China

**Keywords:** supercapacitors, Zn-Cu-Ni oxides, solar energy, photo-enhancement, nanoarrays

## Abstract

To meet the increasing demands of energy consumption, sustainable energy sources such as solar energy should be better employed to promote electrochemical energy storage. Herein, we fabricated a bifunctional photoelectrode composed of copper foam (CF)-supported zinc-nickel-copper ternary oxides in nanoarrays (CF@ZnCuNiO_x_ NAs) to promote photo-enhanced pseudocapacitive charge storage. The as-fabricated CF@ZnCuNiO_x_ NAs have shown both photosensitive and pseudocapacitive characteristics, demonstrating a synergistic effect on efficient solar energy harvest and conversion. As a result, a high areal specific capacitance of 2741 mF cm^−2^ (namely 418 μAh cm^−2^) under light illumination can be calculated at 5 mA cm^−2^, which delivered photo-enhancement of 38.3% compared to that obtained without light. In addition, the photoelectric and photothermal effects of the light energy on pseudocapacitive charge storage have been preliminarily studied and compared. This work may provide some evidence on the different mechanisms of photoelectric/thermal conversion for developing solar-driven energy storage devices.

## 1. Introduction

Energy storage devices, such as metal (e.g., Li, Na, Zn) ion batteries and supercapacitors, are essential in our daily energy supply. Among them, supercapacitors are considered highly promising due to their low cost, high power density, and ultralong cycle life [1,2,3]. Traditional supercapacitor cells include electric double-layer capacitors (EDLCs) that are based on adsorption/desorption characteristics and pseudocapacitors that employ Faradaic reactions for efficient charge storage [4]. However, the current supercapacitor devices manufactured from carbon-based electrode materials are only able to deliver much lower energy densities than those of metal ion batteries, thus largely hindering their future applications in large-scale energy storage [5]. Meanwhile, typical pseudocapacitive electrode candidates such as MnO_2_, NiO, and Co_3_O_4_ usually suffer from irreversible Faradaic reactions, which may lead to server capacitance loss during prolonged cycling tests despite their relatively high theoretical capacities. Luckily, outdoor charging activities are more commonly seen in modern life, which gives a high possibility of employing natural sunlight to further increase the energy density and life span of supercapacitors by external photocharging [6]. In this regard, the design and fabrication of bifunctional photoelectrodes that can harvest solar energy to promote charge storage efficiently are highly compelling and interesting.

Mixed-metal oxides (MMOs, e.g., NiCo_2_O_4_, CoMn_2_O_4_) are considered promising candidates for electrochemical energy devices in lithium ion batteries [7,8,9], supercapacitors [9,10], electrocatalysis [11,12], and sensing [13] due to their low toxicity, high availability, and enriched redox reactions offered by multiple metal ions [14]. In particular, nickel oxides (NiO) and copper oxides (CuO) are two important types of MOs, which have been studied intensively for superior pseudocapacitive performance with high specific capacity and a stable cycle life [15,16,17,18,19]. Meanwhile, zinc oxide (ZnO) is a widely reported photocatalyst with a band gap of ~3.2 eV, which can harvest UV light to generate photogenerated charge carriers [20]. Therefore, the appropriate combination of NiO, CuO, and ZnO may not only improve the pseudocapacitive activity of the as-formed MMOs, but may also promote charge storage capability by offering additional photogenerated charge carriers upon light illumination. Meanwhile, the presence of CuO (Eg ~1.7 eV) may bring about a photothermal effect under light illumination, which could be favorable for improving the charge transfer during the redox reactions. The design approach of metal oxides incorporating intermediate thin film metallic layers has been reported to be effective at improving the performance of MMOs [21,22]. It should also be noted that the construction of unique hierarchical nanostructures for MMO material is also significant to light harvesting because of the random irradiation of incident sunlight.

Herein, we have described a simple method to fabricate CF@ZnCuNiO_x_ NAs as bifunctional photoelectrodes for photo-enhanced supercapacitors. The CF was used as both a copper source and growth substrate for the initial deposition of Cu(OH)_2_ NAs. Subsequently, the Cu(OH)_2_ NAs were uniformly coated with a layer of ZIF-8 precursors to facilitate the deposition of Ni-based precursors. The next deposition of the nickel source led to the formation of ternary Zn-Cu-Ni layered double hydroxides (LDHs) caused by the outward diffusion of copper species. Finally, the ZnCuNiO_x_ MMOs in nanoarrays are constructed by thermal treatment of these Zn-Cu-Ni LDHs in the air. The supercapacitor properties of the as-prepared CF@ZnCuNiO_x_ NAs were then evaluated under light illumination, and circulating cooling water (20 °C) was also equipped during the electrochemical measurements to estimate the photothermal effects. As a result of the synergistic effect of photosensitive and pseudocapacitive characteristics, the as-fabricated CF@ZnCuNiO_x_ photoelectrodes delivered a maximum specific capacitance of 2741 mF cm^−2^ at 5 mA cm^−2^ and a maximum photoenhancement of 61.5% at 30 mA cm^−2^ under light illumination. The as-obtained capacitance of 2741 mF cm^−2^ (418 μAh cm^−2^) is much higher than that of the conventional supercapacitor structure using a similar active redox component reported recently (NiO nanosheets array@Co_3_O_4_-NiO FTNs: Maximum areal capacity of 1217.5 mF cm^−2^/169 μAh cm^−2^ at 5 mA cm^−2^) [23].

## 2. Materials and Methods

### 2.1. Synthesis of CF@Cu(OH)_2_ Nanoarrays

The synthesis procedure followed previous work with modifications [24]. First, 6.4 g of NaOH was dissolved in 16 mL of DI water. Then, 1.826 g of (NH_4_)_2_S_2_O_8_ was dissolved in 44 mL of DI water. Then the former solution was mixed with the latter under vigorous magnetic stirring at room temperature (RT). After 60 s, the stirring ceased, and a piece of CF (1 × 4 × 0.15 cm^3^) was immersed in the mixture before being kept at RT for 25 min. Later, the CF was taken out and washed with water and ethanol several times, and then dried in an air-flow oven at 60 °C overnight.

### 2.2. Synthesis of CF@Cu(OH)_2_@ZIF-8

First, 656.8 mg of 2-methylimidazole (2-mim) was dissolved in 30 mL of methanol (MeOH), while 297.5 mg of Zn(NO_3_)_2_·6H_2_O was dissolved in another 30 mL of MeOH. Next, the former solution was mixed with the latter under vigorous magnetic stirring at RT. After 60 s, the stirring ceased, and a piece of the as-prepared CF@Cu(OH)_2_ film was immersed in the mixture before being kept at RT for 2 h. Finally, the film was taken out and washed with water and ethanol, and then dried in an air-flow oven at 60 °C overnight.

### 2.3. Synthesis of CF Supported Zn-Cu-Ni LDH and ZnCuNiO_x_

First, 72.7 mg of Ni(NO_3_)_2_·6H_2_O was dissolved in 20 mL of MeOH, while 41.1 mg of 2-mim was dissolved in 10 mL of MeOH. Then the former solution was mixed with the latter under vigorous magnetic stirring at RT. After 5 min, a piece of CF@Cu(OH)_2_@ZIF-8 film was immersed in the mixed solution, and then the mixture was transferred into a Teflon-lined stainless-steel autoclave before being heated in an air-flow oven at 150 °C for 6 h. After cooling down to RT, the film (CF@Zn-Cu-Ni LDH) was taken out and washed with DI water and ethanol several times before being dried in an air-flow oven at 60 °C overnight. To derive the CF-supported Zn-Cu-Ni MMOs (ZnCuNiO_x_), the as-obtained CF@Zn-Cu-Ni LDH was annealed in a muffle furnace at 400 °C for 2 h.

### 2.4. Material Characterization and Electrochemical Measurements

The crystal structures of the obtained samples were identified by X-ray diffraction (XRD, Rigaku D/max 2500, Cu Kα radiation, λ = 0.1518 nm, Applied Rigaku Technologies, Inc., Austin, TX, USA). The morphologies and compositions were examined using a scanning electron microscope (SEM, Quanta FEG 250, FEI Company, Hillsboro, OR, USA) equipped with energy-dispersive X-ray spectroscopy (EDX), and a transmission electron microscope (TEM, Titan G2 60-300, FEI Company, Hillsboro, OR, USA) equipped with a mapping system. Specifically, the film sample was ultrasonicated in ethanol for 15 min before the suspension was dropped onto a piece of silicon wafer for the EDX test. The UV–visible diffuse reflectance spectrometry (UV–vis DRS) was performed using a Shimadzu UV-3600 spectrometer (Kyoto, Japan), where BaSO_4_ was used as a reference.

The electrochemical performances of the as-prepared samples were investigated with a three-electrode electrochemical workstation (IVIUM, Vertex. C. EIS, Ivium Technologies, Eindhoven, The Netherlands). The as-prepared material (1 × 1 cm^2^ nominal planar area) was directly used as the working electrode in a 2 M KOH aqueous solution, and a saturated calomel electrode (SCE) was used as the reference electrode (all the potentials mentioned throughout the manuscript are versus SCE), and platinum foil (1 × 1 cm) as the counter electrode. Cyclic voltammetry (CV) and galvanostatic charge/discharge (GCD) were carried out at different voltage scan rates or current densities. The specific areal capacities were calculated from the GCD curve according to the following Equation (1) [25]:

(1) $$C=\frac{I\Delta t}{\mathrm{UA}}\;$$

where C (mF cm^−2^) is the specific areal capacitance, I (A) is the discharge current, Δt (s) is the discharge time, U (V) is the potential window, and A (cm^2^) is the electrode area.

The electrochemical measurements under light illumination were carried out in a sealed quartz glass cell under ambient air pressure. The configuration of the three-electrode electrochemical system remained unchanged. A 300 W xenon arc lamp (Perfect Light PLS-SXE300, Perfectlight Technology, Beijing, China) was used as the light source (light power = 50 W; spotlight diameter = 60 mm). The photocurrent response was performed with an open circuit potential using a switched light on/off mode (the time interval is 60 s). The subsequent CV and GCD results were collected with or without light illumination, respectively. At the same time, circulating cooling water was employed to maintain the temperature of the glass cell at 25 °C to estimate the photothermal effects on the results. Specifically, a glass container with two connected ends was used, inside which a quartz electrolytic cell was placed, and the cooling water was filled until the electrolyte in the cell was submerged. The condensate was kept at a constant temperature of 25 °C, thus maintaining a constant temperature for the electrolytic cell. As for the sample, the size of the electrode was 1 × 1 cm^2^. For the electrochemical measurements, there were three testing cases in this work: (1) Without light illumination or cooling water; (2) with light illumination but no cooling water; (3) with light illumination and cooling water.

## 3. Results and Discussion

**Figure 1:**
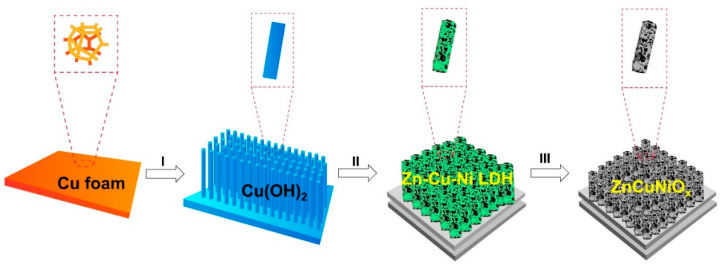
Schematic formation procedure of the CF@Cu(OH)_2_, CF@Cu(OH)_2_@Zn-Cu-Ni LDH, and CF@ZnCuNiO_x_ Nas..

The synthetic procedure of CF@ZnCuNiO_x_ NAs is schematically presented in Figure 1. The CF was used as both the skeleton and copper source for the formation of the CF-supported Zn-Cu-Ni MMOs. In step I, copper hydroxide [Cu(OH)_2_] NAs were vertically grown on the CF skeleton by etching a piece of CF in a NaOH/(NH_4_)_2_S_2_O_8_ aqueous solution. In the next step II, the Cu(OH)_2_ NAs were modified with a layer of ZIF-8 precursor and then used as substrates for the subsequent growth of Ni-based precursors, thus leading to the formation of a Zn-Cu-Ni LDH hierarchical structure. Finally, the as-formed Zn-Cu-Ni LDH precursor was converted into Zn-Cu-Ni MMOs by thermal treatment at 400 °C in the air (step III). It should be noted that the CF skeleton was well retained after the thermal treatment, thus generating the robust CF-supported ZnCuNiO_x_ (CF@ZnCuNiO_x_) NAs. The as-prepared CF@ZnCuNiO_x_ NAs were then directly used as bifunctional photoelectrodes for pseudocapacitive charge storage in photo-enhanced supercapacitors.

Figure S1 (Supplementary Materials) shows the X-ray diffraction (XRD) patterns of CF@Cu(OH)_2_, CF@Cu(OH)_2_@ZIF-8, CF@Cu(OH)_2_@ZIF-8@Zn-Cu-Ni LDH, and CF @ZnCuNiO_x_, respectively. Except for the typical peaks of Cu (JCPDS no. 70-3048) [26] for all the samples, peaks of Cu(OH)_2_ (JCPDS no. 13-0420) [27] can be identified for the former three samples only, indicating a complete conversion of Cu(OH)_2_ to oxides after thermal treatment in the CF@ ZnCuNiO_x_ sample. In addition, peaks of CuO (JCPDS no. 89-5897) [28] and NiO (JCPDS no. 47-1049) [29] can be found in the CF@ZnCuNiO_x_ sample, thus confirming the final formation of Zn-Cu-Ni MMOs. Due to the low content of ZIF-8, the XRD peaks of ZnO were not detected for the CF@ZnCuNiO_x_ sample; however, the presence of ZnO in the as-prepared Zn-Cu-Ni MMOs can still be verified by the later TEM results. To obtain the elemental composition of the final Zn-Cu-Ni MMOs, SEM-EDX was carried out, and the results are displayed in Figure S2. The atomic ratios of Zn, Cu, Ni, C, and N are determined to be 0.31%, 7.44%, 2.15%, 29.71%, and 5.02%, respectively. The presence of C and N should be due to the annealing residue of the organic additives involved during the synthesis. The strong peak of Si is due to the silicon wafer used as the substrate for the test.

**Figure 2:**
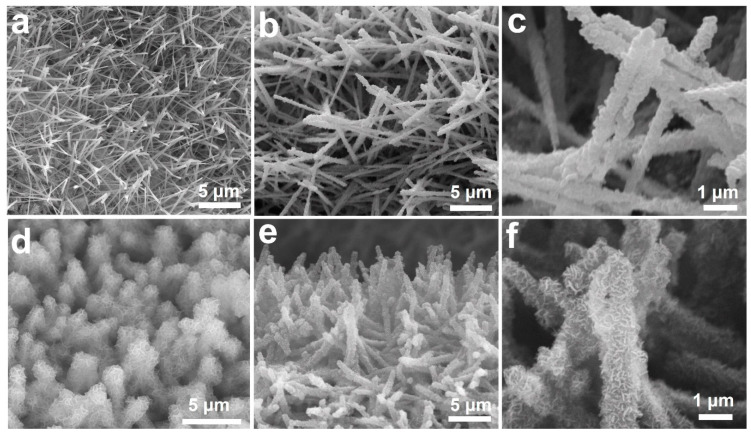
SEM images (**a**–**f**) of Cu(OH)_2_ NAs (**a**), ZIF-8 modified Cu(OH)_2_ NAs (**b**,**c**), Zn-Cu-Ni LDH (**d**), and ZnCuNiO_x_ NAs (**e**,**f**).

The detailed morphology and elemental distribution of the obtained samples were examined by SEM, TEM, HRTEM, and elemental mapping. As shown in Figure 2a, the dense Cu(OH)_2_ NAs composed of nanopillars with an average aspect ratio of ~17 (length ~6 μm, width ~340 nm) are vertically arranged. After surface modification by ZIF-8, the nanopillars were remarkably thickened to ~600 nm, as shown in Figure 2b,c, indicating a coating thickness of approximately 130 nm. With the assistance of ZIF-8, the Ni-based precursors can be readily deposited against the nanopillars to form Zn-Cu-Ni LDH in a hierarchical nanosheets structure (Figure 2d). After the thermal treatment in air, the Zn-Cu-Ni LDH precursors were completely transformed into Zn-Cu-Ni MMOs (ZnCuNiO_x_) with a well-retained nanosheet structure, which can be confirmed by the SEM results in Figure 2e,f. The arrangement of the finally obtained ZnCuNiO_x_ NAs is similar to the pristine Cu(OH)_2_ NAs, revealing robustness of the overall structures.

**Figure 3:**
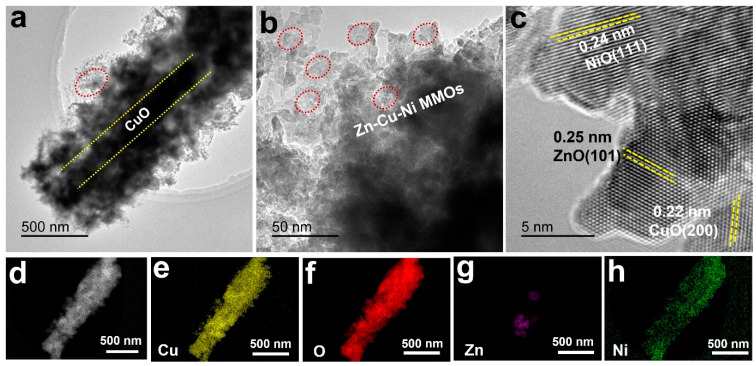
TEM (**a**,**b**), HRTEM (**c**), dark-field TEM (**d**), and elemental mapping (**e**–**h**) images of the ZnCuNiO_x_ MMOs.

The typical TEM image (Figure 3a) and magnified TEM image (Figure 3b) clearly exhibit the core-shell structure of a single ZnCuNiO_x_ nanopillar, confirming the synthetic routes in Figure 1. It is explicit that the nanopillars are encapsulated by hierarchical nanosheets, which may indicate the presence of CuO as a core structure (within the two yellow dotted lines in Figure 3a) and Zn-Cu-Ni MMOs as shells (indicated by the red ovals in Figure 3a,b), respectively. Such a core-shell hierarchical nanostructure may endow the material with more active sites for electrochemical reactions. Although the XRD patterns of CF@ZnCuNiO_x_ NAs have no characteristic peak of ZnO, an HRTEM image (Figure 3c) reveals the typical lattice spacing distances of 0.25, 0.22, and 0.24 nm, which can be indexed to ZnO (101) [30], CuO (200) [31], and NiO (111) [32], respectively. This result suggests that the Zn, Cu, and Ni species were homogeneously mixed in the nanosheets with a lattice-scale interface. Further elemental mapping was used to examine the distribution of all the elements within the nanosheets. Based on the dark-field TEM image shown in Figure 3d, the copper element is uniformly distributed over the whole core-shell nanopillar (Figure 3e), verifying the existence of Cu species in both core and shell structures.

Similarly, the O element is present in both core and shell structure, which shows a coincident distribution with Cu (Figure 3f), verifying the formation of CuO in the core-shell structure. In contrast, the signal intensity of the Zn element (Figure 3g) is much weaker than that of Cu, which is possibly due to the low content of ZnO derived from the thin layer of ZIF-8 (thickness ~130 nm, recall Figure 2c). Remarkably, the distribution area of the Ni species is almost located at the outer structure of the nanopillar (Figure 3h), which suggests that the nanosheets are mainly composed of the NiO component. All the elemental mapping results have again validated that the chemical composition of the nanopillars is Zn-Cu-Ni MMOs with a lattice-scale contact interface. The homogeneous mix of all the MMOs may endow a rapid and efficient transfer of charge carriers during the electrochemical redox reactions [14].

The UV-vis DRS was then employed to confirm their light-harvesting capabilities, and the results are presented in Figure S3. It can be seen clearly that the visible-light absorption (400–650 nm) of the ZnCuNiO_x_ MMOs was largely improved compared to the other three precursor samples, suggesting a good utilization of light energy by the CF@ZnCuNiO_x_ photoelectrode. Before the electrochemical measurements, photocurrent response was tested for the CF@ZnCuNiO_x_ NAs with open circuit potential (Figure S4). A transient photocurrent of −350 μA cm^−2^ was delivered when the light was on, and a steady value of −10 μA cm^−2^ was maintained for the next cycles. When the light was switched off, the current was reversed to 230 μA cm^−2^, and then stabilized at 27 μA cm^−2^ in the next cycles. The quick response of the photocurrent upon the light switching on and off suggests CF@ZnCuNiO_x_ NAs are highly sensitive to light illumination, which results from the photoelectric effect of the MMOs [33,34]. The photogenerated charge carriers may be favorable to promoting the redox reaction during electrochemical energy storage.

**Figure 4:**
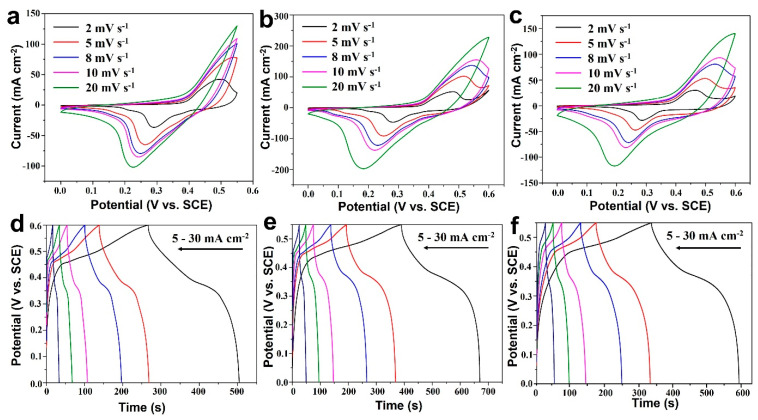
CV (**a**–**c**) and GCD (**d**–**f**) curves of the CF@ZnCuNiO_x_ NAs collected without light illumination (**a**,**d**), under light illumination but without cooling water (**b**,**e**), and with light illumination and cooling water (**c**,**f**).

The electrochemical performances of the as-prepared CF@ZnCuNiO_x_ NAs were evaluated in a three-electrode system under light illumination with/without circulating cooling water (installation shown in Figure S5). Figure 4a–c shows the cyclic voltammetry (CV) curves obtained at various scan rates (2, 5, 8, 10, and 20 mV s^−1^). All the curves show strong redox peaks (oxidation peaks within 0.15–0.35 V and reduction peaks within 0.45–0.55 V), which indicates there are two pairs of redox reactions (Cu^2+^/Cu^+^ and Ni^3+^/Ni^2+^) [35,36]. The deviation of redox peaks from the equilibrium states can be observed, which could be caused by the increase in internal diffusion resistance within the pseudocapacitive electrodes [37]. Besides, the oxidation peak of Cu^2+^/Cu^+^ is likely integrated into the Ni^3+^/Ni^2+^ to form the main oxidation peak. It is interesting that the CV currents show a sequence of b > c > a (a: Without light or cooling water; b: With light but without cooling water; c: With light and cooling water), which clearly established a photoenhancement on the current increase in these three test cases. In addition, a higher current density of b than c is likely due to the photothermal effect of b because the cooling water in c ruled out the photothermal effect on the current change.

The GCD curves at various current densities (5, 8, 10, 15, 20, and 30 mA cm^−2^) are illustrated in Figure 4d–f, where the charge–discharge platforms can be clearly observed for all three test cases. Similar to the CV results, the GCD time of e (with light but without cooling water) is much longer than that of d (without light or cooling water) and f (with light and cooling water) at the respective current densities. This result has again confirmed that the introduction of light into photoelectrodes is favorable for improving the electrochemical charge storage.

**Figure 5:**
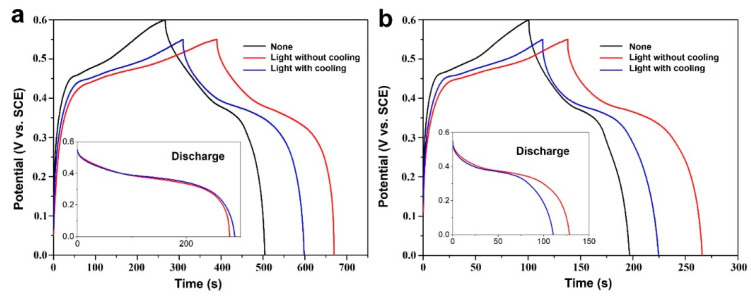
GCD curves collected at a current density of 5 mA cm^−2^ (**a**) and 10 mA cm^−2^ (**b**) of the CF@ZnCuNiO_x_ NAs. The insets indicate the discharge curves obtained at the respective current density.

To further illustrate the photoenhancement on electrochemical charge storage, GCD curves obtained at specific current densities for all three test cases are presented and compared (Figure 5). At a current density of 5 mA cm^−2^, a GCD time of 509 s can be recorded in the test without light or cooling water, while the GCD time was remarkably increased to 672 s under the test case of light illumination (red curve, Figure 5a). When cooling water was applied to the test case of light, the GCD time then decreased from 672 s to 598 s (blue curve, Figure 5a), which could be caused by the removal of the photothermal effect. However, the GCD time is still 89 s longer than that of the test without light or cooling water, which shows that the photoelectric effect may play a key role in photoenhancement.

When the current density was increased to 10 mA cm^−2^, the entire GCD time delivered was reduced for all three testing cases (Figure 5b). Similarly, the testing case of light illumination without cooling water shows the best performance among all the testing cases, which additionally confirms that photoenhancement indeed works for different current densities. Furthermore, it should also be noted that a larger potential window usually leads to a longer GCD time [38]. In our experiment, the testing case without light or cooling water was performed using a potential window of 0–0.6 V, while the latter two cases were conducted with a narrowed potential window of 0–0.55 V as a conservative comparison, which further suggests that the utilization of light energy is an efficient strategy to promote electrochemical charge storage.

**Figure 6:**
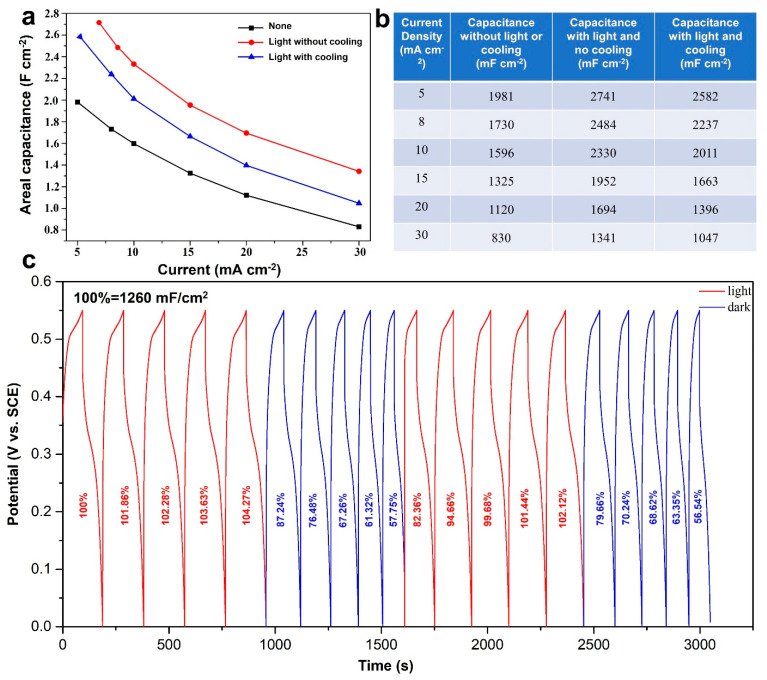
Areal capacitance calculated from various current densities (**a**,**b**) under the three testing cases, and GCD curves obtained at 25 mA cm^−2^ under a switched light on/off mode with cooling water (**c**) of the CF@ZnCuNiO_x_ NAs.

The areal capacitances calculated from various current densities under the three test cases are plotted and summarized in Figure 6a,b. The capacitances obtained from the testing case without light or cooling water ranged from 1981–830 mF cm^−2^ (302–126 μAh cm^−2^) at current densities of 5–30 mA cm^−2^, respectively. Under light illumination, the delivered capacitances at the same current densities are calculated to be 2582–1047 mF cm^−2^ (393–159 μAh cm^−2^) and 2741–1341 mF cm^−2^ (418–204 μAh cm^−2^) with and without cooling water, respectively. Accordingly, high capacitance enhancements of 38.3–61.5% and 30.3–26.1% can be achieved, respectively, for the test cases with and without cooling water. Interestingly, photoenhancement shows a positive relevance to the current densities for the test case without cooling water, while it demonstrates a negative tendency if cooling water is not employed. Such a difference may be caused by the much faster photoelectric process rather than the photothermal effect because it may take more time to heat up the electrode and electrolyte via photo-to-thermal conversion [39,40].

Figure 6c further illustrates the photoelectric effect on charge transfer in the electrochemical process. The GCD curves were collected at a high current density of 25 mA cm^−2^ using a switched light on/off mode in the presence of cooling water (to rule out the photothermal effect). When the light is switched on, the capacitance was increased to 104.2% at its fifth cycle. Then the capacitance retention suddenly dropped to 87.2% once the light was turned off, and further decreased to 57.7% after five successive cycles. Upon light irradiation, the capacitance rapidly jumped back to 82.3%, and then recovered to 102.1% in the fifth cycle. Again, the retention dropped to 79.6% upon switching the light off and further decayed to 56.5% after five cycles. This sharp fluctuation of capacitance retention is highly sensitive to light illumination, which could be attributed to the photoelectric effect of the Zn-Cu-Ni MMOs components. Such photoenhancement including photoelectric and photothermal may provide a new concept to make use of sunlight energy to promote electrochemical energy storage. The morphological change was also examined by SEM characterization, and the SEM images of the CF@ZnCuNiO_x_ NAs after cycling (tested in Figure 6c) are displayed in Figure S6, where the NAs’ structural features with nanosheets can be clearly observed, demonstrating the good structural stability of the as-prepared materials under light illumination. This research work demonstrates a trial exploration of sunlight energy including photoelectric and photothermal effects on pseudocapacitive charge storage using a three-electrode system, and thus may provide some evidence for the development of solar-driven energy storage and conversion devices, such as transparent supercapacitors, lithium/sodium/zinc ion batteries, metal–air batteries, and electrochromic devices [41].

## 4. Conclusions

In this work, a very straightforward synthetic method was employed to prepare hierarchical CF@ZnCuNiO_x_ NAs as bifunctional photoelectrodes for supercapacitors. The as-prepared Zn-Cu-Ni MMOs have demonstrated both pseudocapacitive and photosensitive properties, which can work as bifunctional electrodes for light-energy harvesting and electrochemical charge storage. Significantly, both photoelectric and photothermal effects of the as-prepared photoelectrodes were explored and utilized to promote charge transfer and storage during the pseudocapacitive process. As a result, the high areal capacitance of 2741–1341 mF cm^−2^ (418–204 μAh cm^−2^) can be delivered under light illumination without cooling water compared to those obtained without light, corresponding to a considerable light enhancement of 38.3–61.5%. There is a high potential to use this kind of material to fabricate transparent photoelectrodes for the development of photo-enhanced transparent devices.

## Supplementary Materials

The following supporting information can be downloaded at: https://www.mdpi.com/article/10.3390/nano12142457/s1, Figure S1. XRD patterns of CF@Cu(OH)2, CF@Cu(OH)2@ZIF-8, CF@Cu(OH)2@ZIF-8@Zn-Cu-Ni LDH, and CF@ZnCuNiOx samples, respectively; Figure S2. EDX spectrum of the Zn-Cu-Ni MMOs structure; Figure S3. UV-vis DRS spectra (from curve 1 to 4) of the CF@Cu(OH)2, CF@Cu(OH)2@ZIF-8, CF@Cu(OH)2@ZIF-8@Zn-Cu-Ni LDH, and CF@ZnCuNiOx samples, respectively; Figure S4. Photocurrent response recorded with chopped light irradiation (60 s interval) at an open circuit potential; Figure S5. A photo of the three-electrode installation under light illumination with circulating cooling water; Figure S6. SEM images (a–b) of the CF@ZnCuNiOx NAs after the light on/off cycling test of (c).
