# Supplementary material for: Fabrication of Zn-Cu-Ni Ternary Oxides in Nanoarrays for Photo-Enhanced Pseudocapacitive Charge Storage
Source: Nanomaterials (Basel). 2022 Jul 18;12(14):2457. doi: 10.3390/nano12142457 (PMC9320418; doi:10.3390/nano12142457)
Supplement: Supplementary file 1 [file nanomaterials-12-02457-s001.zip › nanomaterials-1798655-supplementary.pdf]

Article

Fabrication of Zn-Cu-Ni Ternary Oxides in Nanoarrays for Photo-Enhanced Pseudocapacitive Charge Storage

Ruitong Xu [1,†], Jun Pan [2,†], Bo Wu [2], Yangguang Li [1], Hong-En Wang [1,3,4] and Ting Zhu [1,3,4,*]

[1] School of Physics and Electronic Information, Yunnan Normal University, Kunming 650500, China; 2290781440@qq.com (R.X.); 2436391230@qq.com (Y.L.); hongen.wang@outlook.com (H.-E.W.)
[2] School of Materials Science & Engineering, Central South University, Changsha 410083, China; 964229306@qq.com (J.P.); 952068584@qq.com (B.W.)
[3] Yunnan Key Laboratory of Optoelectronic Information Technology, School of Physics and Electronic
Information, Yunnan Normal University, Kunming 650500, China
[4] Key Laboratory of Advanced Technique & Preparation for Renewable Energy Materials, Ministry of Education, Yunnan Normal University, Kunming 650500, China
* Correspondence: zhut0002@ynnu.edu.cn or zhut0002@e.ntu.edu.sg
† These authors contributed equally to this work.

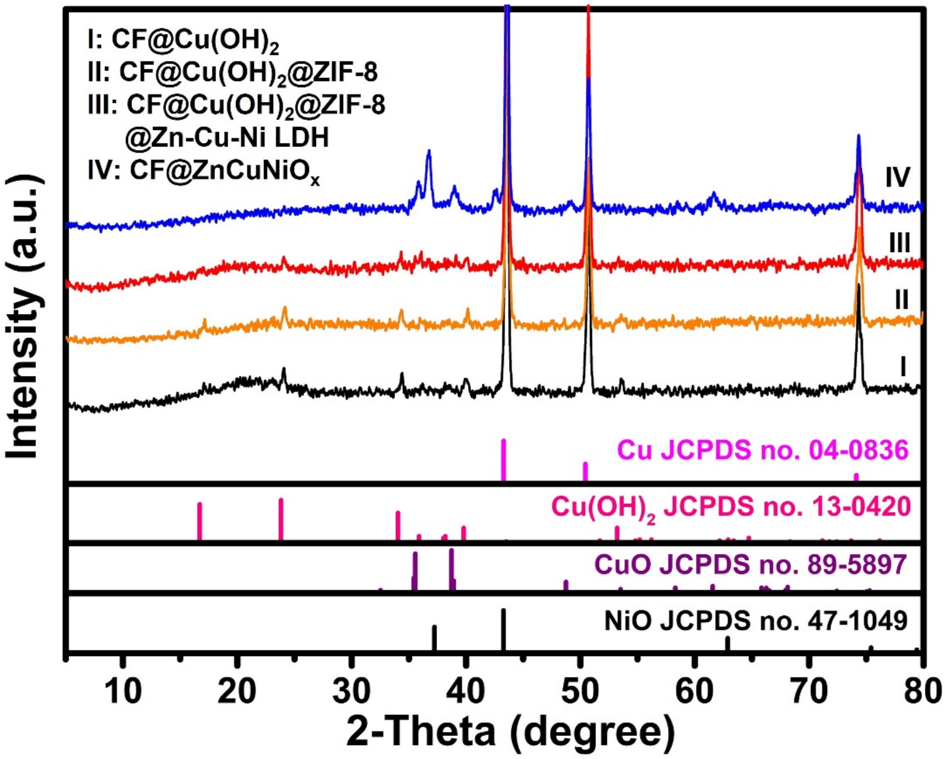

Figure S1. XRD patterns of $CF@Cu(OH)_2$, $CF@Cu(OH)_2@ZIF\text{-}8$, $CF@Cu(OH)_2@ZIF\text{-}8@Zn\text{-}Cu\text{-}Ni$ LDH, and $CF@ZnCuNiO_x$ samples, respectively.

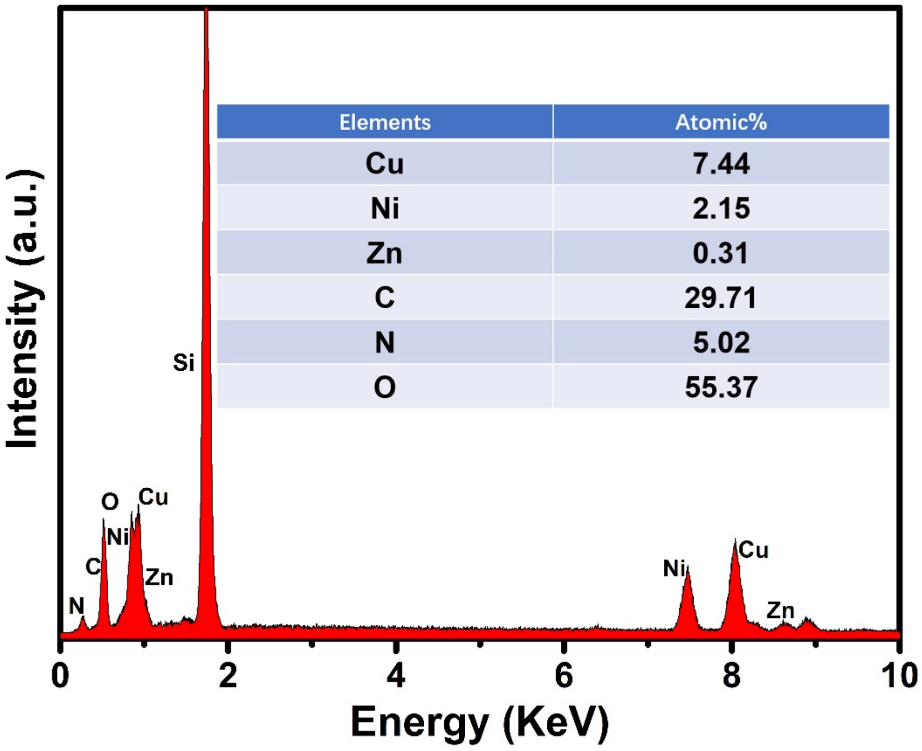

Figure S2. EDX spectrum of the Zn-Cu-Ni MMOs structure.

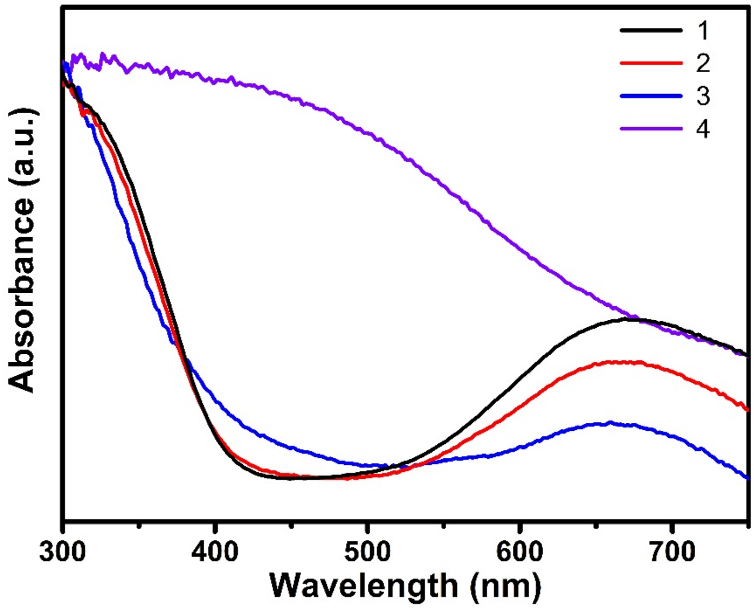

Figure S3. UV-vis DRS spectra (from curve 1 to 4) of the CF@$Cu(OH)_2$, CF@$Cu(OH)_2$@ZIF-8, CF@$Cu(OH)_2$@ZIF-8@Zn-Cu-Ni LDH, and CF@$ZnCuNiO_x$ samples, respectively.

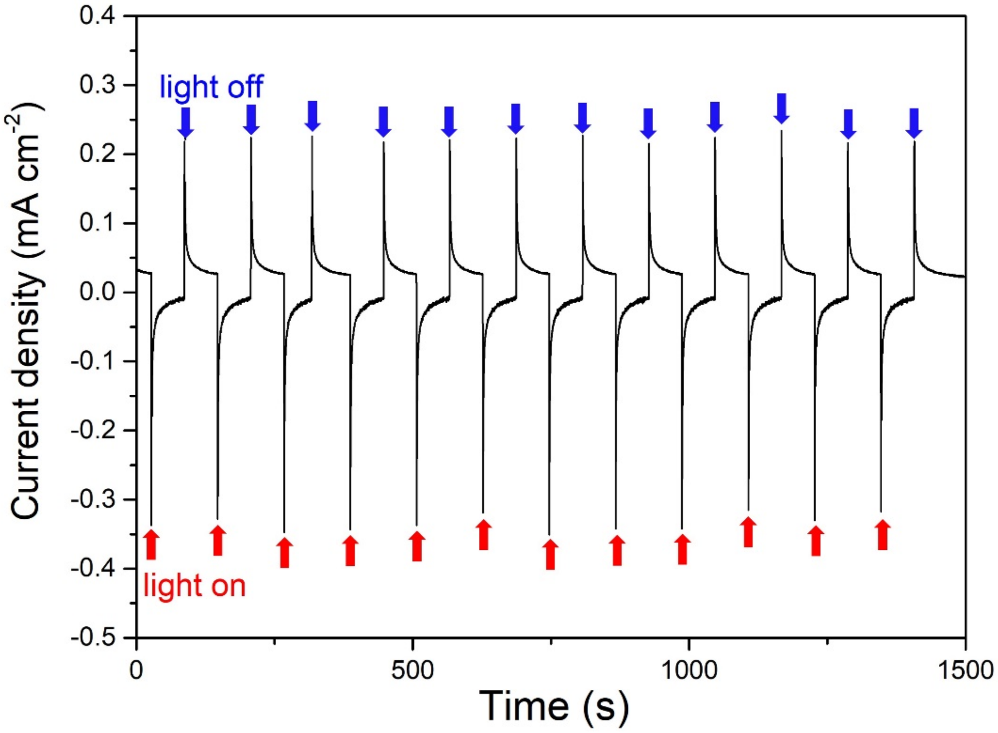

Figure S4. Photocurrent response recorded with chopped light irradiation (60 s interval) at an open circuit potential.

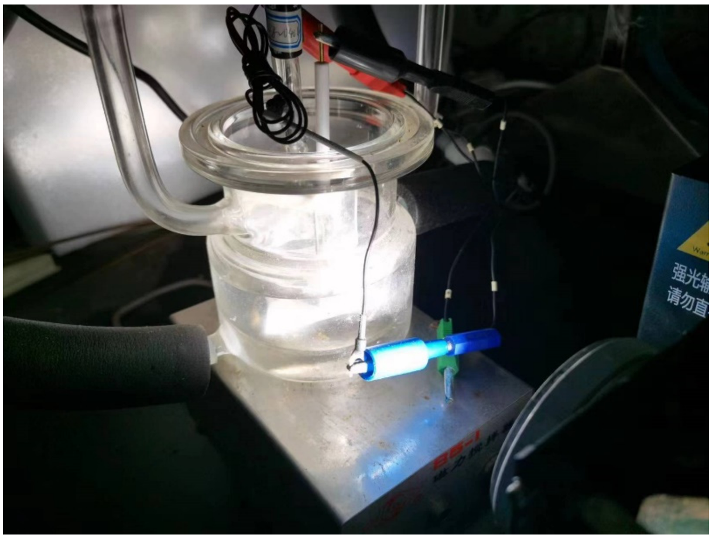

Figure S5. A photo of the three-electrode installation under light illumination with a circulating cooling water.

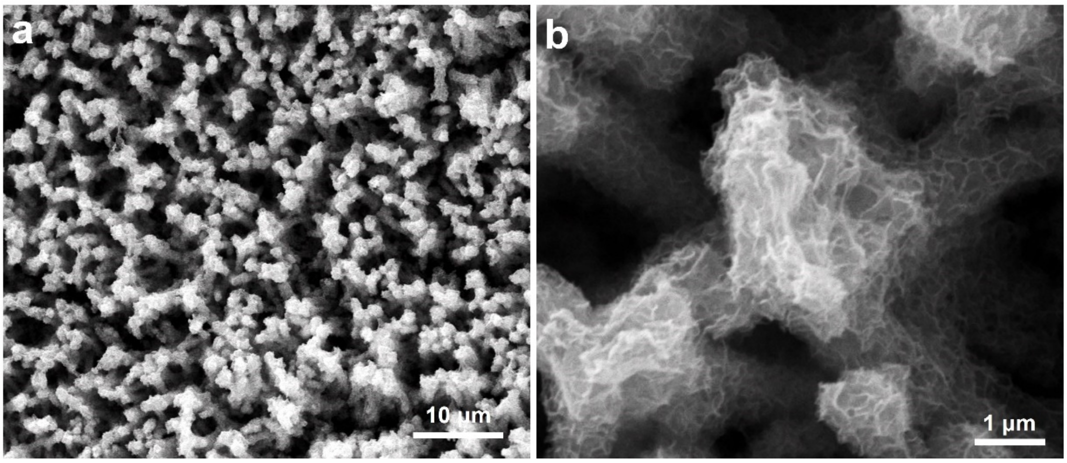

Figure S6. SEM images (a,b) of the $CF@ZnCuNiO_x$ NAs after the light on/off cycling test of Figure 6c.
